# Linear Quantitative Profiling Method Fast Monitors Alkaloids of Sophora Flavescens That Was Verified by Tri-Marker Analyses

**DOI:** 10.1371/journal.pone.0161146

**Published:** 2016-08-16

**Authors:** Zhifei Hou, Guoxiang Sun, Yong Guo

**Affiliations:** 1 School of Pharmacy, Shenyang Pharmaceutical University, Shenyang, China; 2 Department of Pharmaceutical Engineering, Hebei Chemical and Pharmaceutical College, Shijiazhuang, China; 3 School of Pharmacy, Fairleigh Dickinson University, Florham Park, New Jersey, United States of America; Waseda University, JAPAN

## Abstract

The present study demonstrated the use of the Linear Quantitative Profiling Method (LQPM) to evaluate the quality of Alkaloids of Sophora flavescens (ASF) based on chromatographic fingerprints in an accurate, economical and fast way. Both linear qualitative and quantitative similarities were calculated in order to monitor the consistency of the samples. The results indicate that the linear qualitative similarity (LQLS) is not sufficiently discriminating due to the predominant presence of three alkaloid compounds (matrine, sophoridine and oxymatrine) in the test samples; however, the linear quantitative similarity (LQTS) was shown to be able to obviously identify the samples based on the difference in the quantitative content of all the chemical components. In addition, the fingerprint analysis was also supported by the quantitative analysis of three marker compounds. The LQTS was found to be highly correlated to the contents of the marker compounds, indicating that quantitative analysis of the marker compounds may be substituted with the LQPM based on the chromatographic fingerprints for the purpose of quantifying all chemicals of a complex sample system. Furthermore, once reference fingerprint (RFP) developed from a standard preparation in an immediate detection way and the composition similarities calculated out, LQPM could employ the classical mathematical model to effectively quantify the multiple components of ASF samples without any chemical standard.

## Introduction

The dried root of *Sophora flavescens* Ait., known as KuShen (*Sophorae Flavescentis Radix*) in China, is widely used in Traditional Chinese Medicine (TCM) for its effect of clearing heat and dampness, killing parasites, and inducing diuresis [1]. Modern research has also shown various pharmacological effects of Alkaloids of Sophora flavescens (ASF), such as antimicrobial, anti-inflammatory, anti-allergic, anti-tumor, anti-arrhythmia, anti-hepatitis, and regulation of the immune system [2–17]. Among many chemical components from the ASF, quinolizidine alkaloids such as matrine (MT), sophoridine (SPR) and oxymatrine (OMT) have been demonstrated to be important active compounds [18–29]. The quality of ASF as herbal medicine is typically evaluated and controlled by the total alkaloid content. In fact, the National Drug Standard for ASF in China is based on determining the total alkaloid content using a titration method [30]. In practice, the quality of ASF is typically evaluated based on the quantitative content of the marker compounds (e.g., MT, OMT and SPR) determined by high-performance liquid chromatography (HPLC) or capillary electrophoresis (CE) method [31, 32]. But quantitative determination of multiple marker compounds requires significant amount of efforts and resources. In addition to quantitative analysis of the marker compounds, chromatographic fingerprint has been widely adopted for quality assessment of herbal medicines by regulatory agencies, such as US Food and Drug Administration (FDA), European Medicines Agency (EMA), and China Food and Drug Administration (CFDA) [33]. However, the chromatographic fingerprints are commonly analyzed for qualitative similarity among the test samples. The quantitative contents of the chemical components in herbal medicines are not analyzed to compare the similarity of the samples. For example, Zhang et al used a qualitative hierarchical clustering method to analyze the chromatographic fingerprints of the alkaloids from commercial *Sophorae Flavescentis Radix*, and also determined the content of multiple marker compounds for the purpose of quality assessment [34]. They confirmed the usefulness of chromatographic fingerprinting in quality evaluation of herbal medicines, but also acknowledged that direct quantitation of multiple marker compounds was not always feasible due to the availability of the standards for the marker compounds.

The chromatographic fingerprints of the herbal medicine samples are typically used in identification and authenticity [35–36], which only indicates the qualitative similarity in the presence and distribution of the chemical components. The difference in the quantity of the chemical components is not addressed by qualitative similarity analysis. But this is not the case currently. Quantitative similarity analysis has been developed as a measure to detect the difference in the quantitative contents of the herbal samples and has been successfully applied to quality evaluation of some traditional Chinese medicines [37–39]. In this study, the Linear Quantitative Profiling Method (LQPM) was first used to evaluate both linear qualitative and quantitative similarities of the chromatographic fingerprints of the ASF samples. Three alkaloid marker compounds (MT, SPR and OMT) were also quantitated using a validated HPLC method, and the relationship between the linear quantitative similarity (LQTS) and the content of the marker compounds was also investigated. LQPM is a low consumed and highly effective way compared with any multi-marker quantitatively determination method. As a complex multiple component system, ASF samples can be firstly identified and secondly quantified by LQPM from an overall mode. Quality consistency of ASF samples has been successfully monitored and effectively proved by our research results.

## The Linear Quantitative Profiling Method

$\vec{X}=\left(x_{1},x_{2},\cdots ,x_{n}\right)$ and $\vec{Y}=\left(y_{1},y_{2},\cdots ,y_{n}\right)$ serve as the sample and reference vector where *x*_*i*_ and *y*_*i*_ are the *i*th peak area in the sample fingerprint (SFP) and reference fingerprint (RFP), respectively. The correlation coefficient ***r*** for linear equation $\vec{X}=a+b\vec{Y}$ can be calculated according to Eq 1 and represents a linear qualitative similarity (LQLS), which describes the distribution characteristics of all chemical fingerprints in the herbal medicines. The slope ***b***, calculated according to Eq 2, can be used to quantitatively compare $\vec{X}$ and $\vec{Y}$ after correction of the apparent weight of SFP (*m*_*j*_) and RFP (*m*_*R*_). The parameter *m*_*j*_ is the weight of the *j*th sample and the parameter *m*_*R*_ is the average weight of 27 batches of samples. Therefore, the slope ***b*** is called as linear quantitative similarity (LQTS) and can be used to measure quantitative similarity in the total content of all fingerprint components between SFP and RFP. In fact, ***b*** is very close to the apparent content similarity ***R***% (seen in Eq 3), and the error ***e*** (be equal to $=R-b=a{\left(\bar{y}\right)}^{-1}\times 100\%$) is about less than 3%. The term *α* is a statistical error calculated according to Eq 4 and reflects the accuracy of the linear model. The linear qualitative similarity (***r***), linear quantitative similarity (***b***) and error term (*α*) are combined in the Linear Quantitative Profiling Method (LQPM) to evaluate the quality of the herbal medicines (8 grades listed in Table 1).

**Table 1:** The quality grade assigned by LQPM and the acceptance criteria.

Quality grade	1	2	3	4	5	6	7	8
	best	better	good	fine	moderate	common	inferiors	defective
***r***≥	0.95	0.90	0.85	0.80	0.70	0.60	0.50	<0.50
***b***∈	95∼105	90∼110	85∼115	80∼120	70∼130	60∼140	50∼150	0∼∞
***α***≤	0.05	0.10	0.15	0.20	0.30	0.40	0.50	>0.50

In fact, ***r*** is always less than unity, however ***b*** can be in range of 0—∞, thus there is nearly an orthogonality correlation between ***r*** and ***b***, which indicates that the LQTS assess is more important than LQLS. In addition, as responses of different detectors are dissimilar to various fingerprint components, weight correction factor should be taken into consideration. However, the weight correction factor can be ignored for the homologue fingerprints lying in profiles. The composition similarities of LQLS and LQTS can be used to analyse the contribution of some fingerprint peaks. They can be calculated out at once when one fingerprint peak area was input into the numerator in Eqs 1 and 2, respectively, while the denominator were unchanged. Therefore both LQLS and LQTS robustly construct the two dimentional orthogonal similarities for reasonably evaluating fingerpring profiling of TCM and herbal medicines.

$$r=\frac{\sum_{i=1}^{n}\left(x_{i}-{\vec{x}}_{i}\right)\left(y_{i}-{\vec{y}}_{i}\right)}{\sqrt{\sum_{i=1}^{n}{\left(x_{i}-{\vec{x}}_{i}\right)}^{2}}\sqrt{\sum_{i=1}^{n}{\left(y_{i}-{\vec{y}}_{i}\right)}^{2}}}$$ (1)

$$b=\frac{n\sum_{i=1}^{n}x_{i}y_{i}-\sum_{i=1}^{n}x_{i}\sum_{i=1}^{n}y_{i}}{n\sum_{i=1}^{n}y_{i}^{2}-\sum_{i=1}^{n}y_{i}{}^{2}}\times \frac{m_{R}}{m_{j}}\times 100\%\approx \;\frac{\bar{x}}{\bar{y}}\times \frac{m_{R}}{m_{j}}\times 100\%=R\times \frac{m_{R}}{m_{j}}\times 100\%$$ (2)

$$R\text{\%}=\frac{\sum_{i=1}^{n}x_{i}}{\sum_{i=1}^{n}y_{i}}\times \frac{m_{R}}{m_{j}}\times 100\%$$ (3)

$$\alpha =\left|\frac{a}{b\bar{y}}\right|=\left|\frac{R}{b}-1\right|=\left|\frac{e}{b}\right|$$ (4)

## Materials and Methods

### Reagents and chemicals

**Fig 1:**
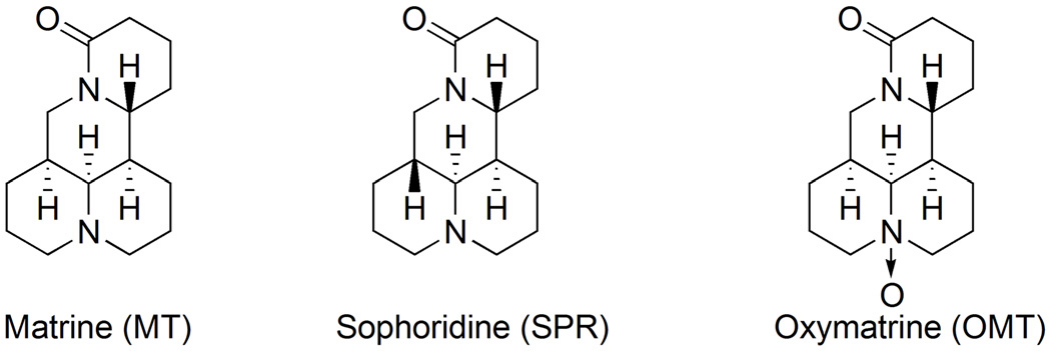
The structures of the marker compounds.

Acetonitrile (HPLC grade) was purchased from Yuwang Industry Co., Ltd (Shandong, China). Phosphoric acid (HPLC grade) was obtained from Kermel Chemistry Reagent Co., Ltd (Tianjin, China) and anhydrous ethanol (HPLC grade) from Fuyu Fine Chemical Co., Ltd (Tianjin, China). De-ionized water and other reagents were of analytical grade. A total of 27 batches of ASF samples (S1-S27) were self extracted in laboratory. The reference sample (RS) of ASF (Batch No. 20151101) was provided by Guangxi HuaHong Pharmaceutical Co. Ltd. Matrine (MT, Batch No. MUST-13021904, purity > 99.2%) and Oxymatrine (OMT, Batch No. MUST-13021902, purity > 99.5%) were provided by Chengdu ManSite Biological Technology Co. Ltd. (Chengdu Institute of biological, Chinese Academy of Sciences). Sophoridine (SPR, batch No.141029, purity>98%) was purchased from Shanghai Winherb Science and Technology Inc. (Shanghai, China). The structures of the three marker compounds are shown in Fig 1.

### Extraction procedure for Alkaloids of Sophora flavescens samples

The dried root of *Sophora flavescens* Ait. was pulverized into powder, and then acidic water was percolated into each powder sample. The percolating liquid was concentrated and the pH was adjusted to between 10 and 11 with a base; then, the liquid was extracted using dichloromethane. The extracted liquid was concentrated by recovering the dichloromethane, and the residue was dissolved in ethanol. ASF samples were obtained after evaporation of the ethanol.

### Instruments and chromatographic conditions

Chromatographic analysis was performed on an Agilent 1100 HPLC series (Agilent Technology, USA), equipped with a diode array detector, a low pressure mix quaternary pump, an online degasser and an auto sampler. Data acquisition was controlled by the ChemStation workstation (Agilent Technology). The chromatographic separation was carried out on an Agilent ZOBAX NH_2_ column (250 × 4.6 mm, 5.0 μm) thermostated at 35°C. The mobile phase was composed of acetonitrile, anhydrous ethanol and water (82:10:8, v/v/v, containing 0.24% phosphoric acid). Isocratic elution was employed at the flow rate of 1.0 mL/min. The injection volume was set at 20 μL. The detection wavelength was set at 210 nm.

Chromatographic fingerprints were processed by an in-house developed software, Digitized Evaluation System for Super-Information Characteristics of TCM Chromatographic Fingerprints 4.0 (Software certificated NO. 0407573, China). SPSS 16.0 and SIMCA 13.0 were also used for data analysis.

### Sample and standard solution preparation

Stock standard solutions were prepared by accurately weighing 9.0 mg, 8.0 mg and 8.0 mg of MT, OMT and SPR standards into separate volumetric flasks of 50 ml, 50 ml, 25 ml respectively. The reference standard was dissolved in adequate ethanol and then diluted to volume with ethanol and stored at 4°C for subsequent use. The mixed standard solution was prepared by pipetting 1.0 ml, 0.5 ml and 1.0 mL of the MT, OMT and SPR stock standard into a 50 ml volumetric flask and then diluting to volume with ethanol.

Approximately 0.10 g of the ASF sample was weighed into a conical flask, and 25.0 mL of ethanol was added to the flask. After the conical flask was capped, the whole flask with the content was accurately weighed. Then the flask was sonicated for 15 minutes (power 320 w, frequency 40 KHZ). After cooling, the flask was weighed again and any lost ethanol was replenished. After filtration, 1.0 mL of the filtrate was pipetted into a 50 mL volumetric flask and diluted to volume with the mobile phase. The sample solution was filtered through 0.45 μm Millipore filters prior to HPLC analysis.

## Results and Discussion

### Quantitation of the three marker compounds

#### Method validation of quantitative analysis

**Fig 2:**
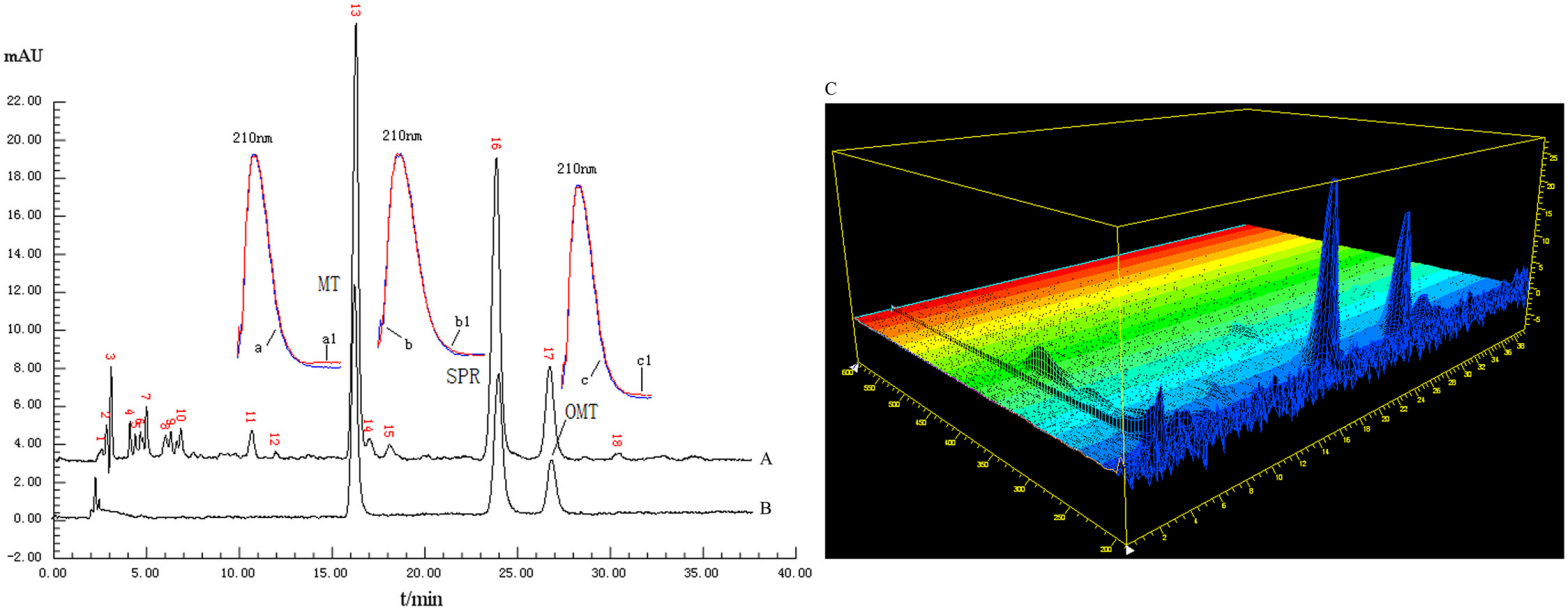
Representative chromatograms of an ASF sample (A), the mixed standards (B) and the 3D spectrum (C). The on-line UV spectra of MT, SPR and OMT (a, b, c for standard and a1, b1, c1 for sample) are shown next to the chromatographic peaks.

Reversed-phase liquid chromatography (RPLC) is typically used for the analysis of ASF. However, quinolizidine alkaloids (e.g., MT, SPR and OMT) are very polar especially when they are protonated. This leads to insufficient retention and peak tailing on the RPLC columns. The present study employed an amino column to separate the chemical components in ASF in the hydrophilic interaction chromatography (HILIC) mode. As shown in Fig 2, the three marker compounds were well retained and separated with very good shape. In addition, other minor components were also eluted mostly early in the chromatogram. The assignment of MT, SPR and OMT was carried out by comparing the retention times and on-line UV spectra with those of standards.

The HPLC method was validated for linearity, system repeatability, accuracy, limit of detection (LOD) and limit of quantitation (LOQ) in support of its application to quantitative analysis of the three marker compounds. The linearity of the HPLC method was assessed at six concentration levels of the three marker compounds as described in Table 2. The calibration curves were established by plotting the peak area versus the injected mass (μg) of the standard marker compounds. Acceptable linearity was demonstrated for the three marker compounds in the concentration range suitable for sample analysis as shown in Table 2. System repeatability was evaluated by the peak area of the three marker compounds following consecutive injections of the mixed standard solution. The relative standard deviation (RSD) was found not to exceed 0.62, 1.15 and 1.10% (n = 6) for MT, OMT and SPR, respectively. The accuracy of the HPLC method was determined by recovery using the standard addition method. The mean recovery of the three marker compounds was between 98.4% and 100.1%, suggesting acceptable accuracy of the method. The LOD and LOQ were determined by appropriately diluting the mixed standard solutions and the results are summarized in Table 2.

**Table 2:** Correlation coefficient (*r*), linear range, LOD and LOQ for the tri-marker compounds.

Compound	Regression eqution	*r*	LOD ^b^ /*n*g	LOQ ^c^ /*n*g	Linear range/μg
**MT**	y = 1256.8*x*-12.181 ^a^	0.999998	2.1	6.3	0.225~1.800
**SPR**	y = 1240.0*x-*17.252	0.999993	3.3	10.0	0.200~1.600
**OMT**	y = 1124.5*x-*3.042	0.999991	0.83	2.5	0.090~0.720

^a^ y and x were, respectively, the peak areas and masses (μg) of the analytes.

^b^ LOD: limit of detection (*S*/*N* = 3)

^c^ LOQ: limit of quantitation (*S*/*N* = 10)

#### Sample analysis

The content of MT, OMT and SPR in ASF samples and one reference sample (RS) was determined using the validated HPLC method, and the results are reported in milligrams per gram of ASF in Table 3. Some variation in the content of each alkaloid was observed in the samples that were tested. For example, the highest contents of MT, SPR, OMT and the total amount (SUM) of them were in S14, meanwhile, the lowest contents of MT, SPR, OMT and the sum of them were in S19.

**Table 3:** The results of the quantitative analysis for the tri-marker compounds and the fingerprint analysis assessed by LQPM.

No.	Content (mg·g^-1^)				*P*_3C_%	*R*%	*ΔE*_1_	*r*	*b*	*ΔE*_2_	*ΔE*_3_	*α*	Grade	Quality
	MT	SPR	OMT	SUM										
**S1**	217.57	231.16	88.55	537.28	96.7	95.5	-1.2	0.997	94.9	-1.9	-0.7	0.007	2	better
**S2**	195.11	201.96	75.97	473.04	84.7	86.6	1.9	1.000	86.1	1.4	-0.5	0.006	3	good
**S3**	235.51	248.77	91.73	576.01	102.9	103.2	0.3	1.000	102.9	0.0	-0.3	0.003	1	best
**S4**	236.03	245.87	90.70	572.59	102.2	102.7	0.5	1.000	102.6	0.4	-0.1	0.000	1	best
**S5**	232.14	243.02	89.59	564.75	100.8	103.4	2.6	1.000	102.2	1.4	-1.3	0.012	1	best
**S6**	254.64	263.75	96.80	615.19	109.7	110.5	0.8	1.000	110.2	0.5	-0.4	0.003	3	good
**S7**	234.36	242.98	94.24	571.58	102.9	103.7	0.8	1.000	102.4	-0.5	-1.3	0.013	1	best
**S8**	240.58	249.81	92.05	582.44	103.9	104.8	0.9	1.000	104.2	0.3	-0.7	0.006	1	best
**S9**	257.31	266.62	98.57	622.51	111.1	111.9	0.8	1.000	111.0	-0.1	-1.0	0.009	3	good
**S10**	244.63	256.93	92.02	593.59	105.5	105.3	-0.2	1.000	105.3	-0.3	0.0	0.000	2	better
**S11**	237.67	246.59	92.77	577.03	103.3	103.5	0.2	1.000	103.6	0.3	0.1	0.001	1	best
**S12**	259.75	271.07	101.86	632.67	113.3	113.7	0.4	1.000	112.6	-0.7	-1.1	0.010	3	good
**S13**	251.95	264.00	94.70	610.65	108.5	107.4	-1.1	1.000	107.2	-1.3	-0.2	0.002	2	better
**S14**	261.54	271.06	104.91	637.51	114.7	113.7	-1.0	1.000	112.6	-2.1	-1.1	0.009	3	good
**S15**	221.87	230.42	84.41	536.70	95.7	97.4	1.7	1.000	96.2	0.5	-1.1	0.012	1	best
**S16**	250.64	260.95	98.17	609.76	109.2	106.9	-2.3	1.000	107.0	-2.3	0.0	0.000	2	better
**S17**	191.30	234.39	85.53	511.22	92.2	92.1	-0.1	0.996	91.6	-0.6	-0.6	0.006	2	better
**S18**	217.96	269.92	97.40	585.28	105.4	105.0	-0.4	0.996	104.4	-1.0	-0.5	0.005	1	best
**S19**	180.07	173.33	63.34	416.74	73.8	75.1	1.3	0.998	76.5	2.7	1.4	0.018	4	fine
**S20**	255.65	244.82	92.22	592.70	105.5	103.3	-2.2	0.998	104.1	-1.4	0.7	0.007	1	best
**S21**	245.99	260.49	98.66	605.14	108.6	104.3	-4.3	0.999	106.0	-2.6	1.7	0.017	2	better
**S22**	231.75	243.71	90.84	566.30	101.3	101.5	0.2	0.999	103.8	2.5	2.3	0.022	1	best
**S23**	220.09	231.14	86.29	537.52	96.2	93.0	-3.2	0.999	94.2	-2.0	1.1	0.012	2	better
**S24**	211.85	223.33	83.54	518.72	92.9	91.9	-1.0	0.999	93.0	0.1	1.0	0.011	2	better
**S25**	215.84	225.80	86.72	528.36	95.0	92.7	-2.3	0.998	92.8	-2.2	0.2	0.001	2	better
**S26**	202.66	211.18	78.75	492.59	88.1	84.6	-3.5	0.998	85.7	-2.4	1.1	0.012	3	good
**S27**	197.52	218.35	78.83	494.70	88.4	88.5	0.1	0.998	89.3	0.9	0.8	0.009	3	good
**RFP**	228.77	240.79	89.50	559.06	100.0	100.0	0.0	1.000	100.0	0.0	0.0	0.000	1	best
**RS**	228.63	237.62	86.59	552.84	98.5	98.8	0.3	0.998	99.3	0.8	0.5	0.005	1	best

**Fig 3:**
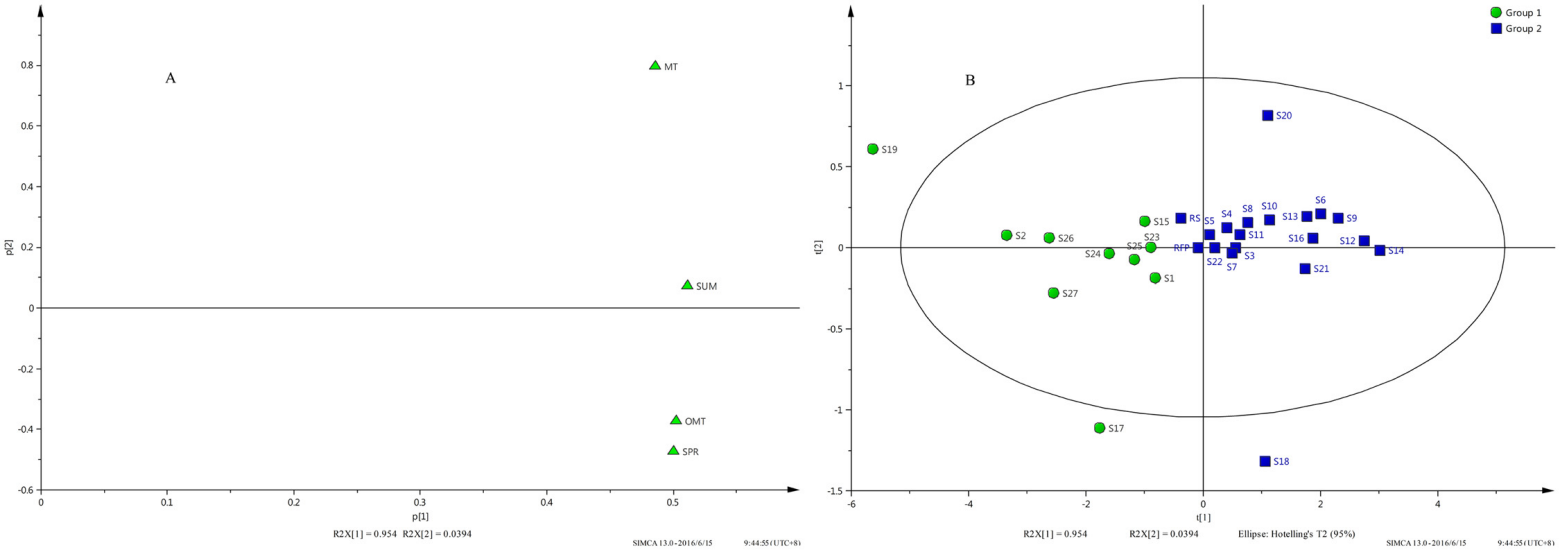
The PCA loading plot (A) and scores scatter plot (B) for all the ASF samples.

In order to evaluate the discriminating ability of the marker compounds, principle component analysis (PCA) was performed using the individual contents of the marker compounds and the total amount (SUM) as the input data to construct a two-dimensional matrix with 29 observations and 4 variables (29x4). As shown in the loading plot (Fig 3A), all four variables are positively correlated to PC1 with the sum of three marker compounds having the highest loading on PC1. In contrast, only MT is positively correlated to PC2 significantly and OMT and SPR have negative loadings on PC2. The two-component PCA model accounts for 95.40% and 3.94% of the variation in PC1 and PC2, respectively.

The score scatter plot (Fig 3B) shows that all the samples are grouped into two clusters marked by Group1 and Group 2, respectively. The samples in Group 1 (S1, S2, S15, S17, S19, S23, S24, S25, S26 and S27) all have negative values on PC1, indicating that the content of the marker compounds were all relatively lower (in the ranges of 180.07–221.87 mg/g for MT, 173.33–234.39 mg/g for SPR, 63.34–88.55 mg/g for OMT and 416.74–537.52 mg/g for SUM, respectively). The samples in Group 2 (S3-S14, S16, S18, S20, S21 and S22) with positive values on PC1 have relatively higher content of the marker compounds (in the ranges of 217.96–261.54 mg/g for MT, 243.02–271.07 mg/g for SPR, 89.59–104.91 mg/g for OMT and 564.75–637.51 mg/g for SUM, respectively). Among 27 samples, only three samples (S17, S18 and S19) were found to be outliers (outside the ellipse) based on the content of the marker compounds. It is also interesting to note that the reference sample (RS) with a negative value on PC1 and the reference fingerprint (RFP) are situated very close to the origin, indicating the “synthesized” reference fingerprint (RFP) is very similar to an independently acquired reference sample.

### Chromatographic fingerprint analysis

#### Method validation of fingerprint analysis

The peak of MT was assigned and selected as the reference peak (shown in Fig 2), then the relative retention time and the relative peak area can be calculated out. The instrument precision was 6 replicated loading S1 sample solution to determine the average qualitative similarity (AQLS) of 0.963 (RSD = 3.5%) and the average quantitative similarity (AQTS) of 100.5% (RSD = 0.73%). The sample solution stability was analyzed once every 95 minutes after prepared within 11 hours and was found stable with AQLS of 0.978 (RSD = 1.75%) and AQTS of 100.2% (RSD = 1.31%). The method repeatability was assessed by analyzing six independently prepared samples (S1) by above analytical procedures to give AQLS of 0.976 (RSD = 0.75%) and AQTS of 100.1% (RSD = 0.83%). The above AQLS and AQTS were calculated out by the self-made TCM fingerprint software. The validation results exhibited that the method satisfied the fingerprint analysis criteria.

#### Evaluating ASF quality by LQPM based on the fingerprint profiling

**Fig 4:**
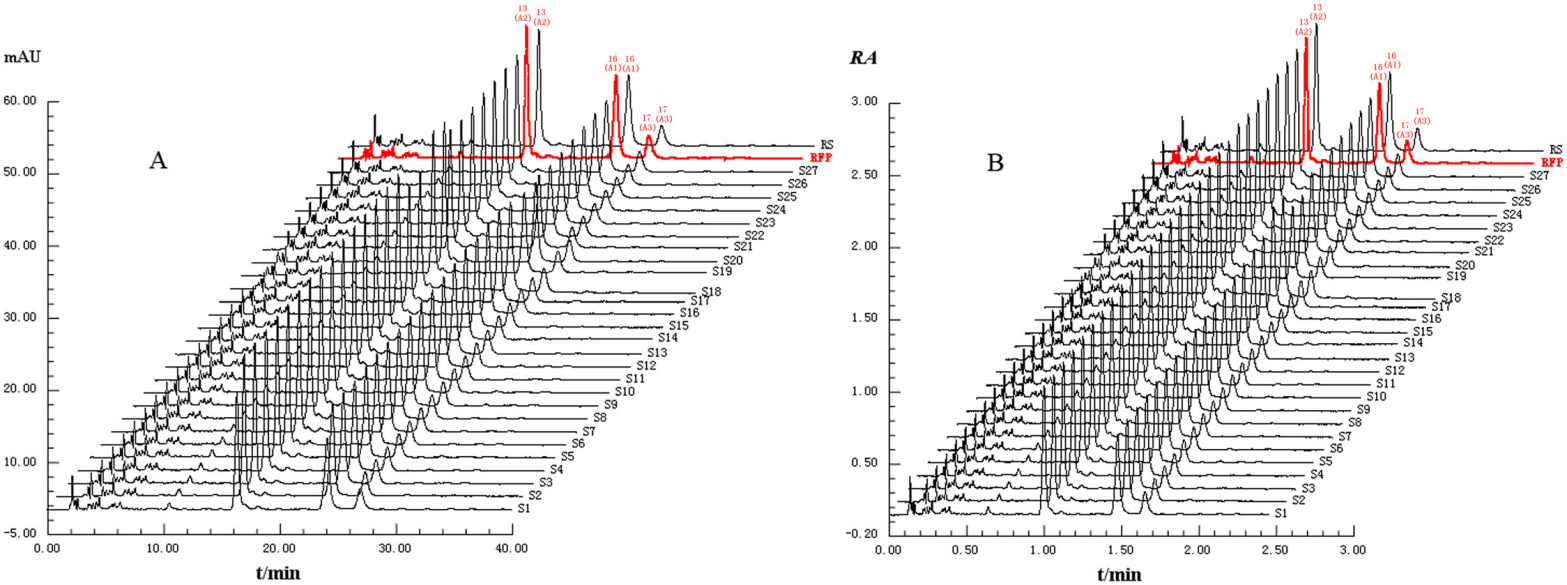
The HPLC fingerprints of 27 batches of the ASF samples, the reference fingerprint (RFP), and the reference sample (RS) detected at 210 nm. (A) Normal HPLC fingerprints (B) the relative characteristic profilings referenced by peak MT.

The validated HPLC method was used to generate chromatographic fingerprints of the ASF samples (shown in Fig 4A). At the analytical wavelength of 210 nm, 18 common peaks were found in all samples and the amplified chromatogram was given with labeled peak numbers in Fig 2A. The reference fingerprint (RFP) was constructed as the authentically fingerprint by averaging all the 27 sample fingerprints. The relative characteristic fingerprint (RCFP, shown in Fig 4B) was constructed by plotting the relative peak area versus the relative retention time. The sample fingerprints and reference fingerprint were imported to the in-house software to calculate the assessing results as presented in Table 3. The results show that all the samples have linear qualitative similarity (***r***) higher than 0.996 and error term *α* ≤ 0.022, indicating that all the samples are similar in the distribution of chemical components. In comparison, the linear quantitative similarity (***b***) has a wider range (76.5–112.6%). As a LQTS measure, ***b*** can exactly discriminate the samples from total contents of all fingerprint peaks but actually ***r*** is disabled for the function. For example, S19 has lower linear quantitative similarity (***b***) (76.5%), although the linear qualitative similarity (***r***) is very high (0.998). This indicate that ***b*** is a useful discriminating tool to differentiate the ASF samples when ***r*** is very close to unity.

In terms of the criteria (shown in Table 1), the qualities of S3, S4, S5, S7, S8, S11, S15, S18, S20, S22 and RS were best (Grade 1), those of S1, S10, S13, S16, S17, S21, S23, S24 and S25 were better (Grade 2), and those of S2, S6, S9, S12, S14, S26 and S27 were good (Grade 3), except for that of S19 as fine (Grade 4) due to the much lower contents for the 18 components. In comparison, the PCA method identified three samples (S17, S18 and S19) as outliers, and S18 was singled out because the relative amounts of the marker compounds are much higher than other samples. However, it does not necessarily mean that S18 has poor quality. In fact, S18 has acceptable quality (Grade 1) based on the quantitative fingerprint analysis. In addition, two more samples were identified as dissimilar (S17 belonged to Grade 2 and S19 belonged to Grade 4) based on different linear quantitative similarities.

This observation highlights the point that PCA can be over discriminating in some cases and the quantitative fingerprint analysis can provide a more accurate evaluation of the herbal samples.

#### Investigating the composition similarity of ASF reference fingerprint

According to ASF reference fingerprint, we can calculate the composition similarity of the three biggest peaks i.e. peak16, peak13 and peak17 with 0.478, 0.458, 0.055 of ***r***, and 36.9%, 35.8% and 12.6% of ***b***, respectively. The total value of ***r*** for the tri-marker is 0.99 and the total value of ***b*** for the tri-marker is 85.3%, which clearly express for the dominant contribution, respectively. So the ASF quality is absolutely occupied by the tri-marker but the other lower contents of components can be controlled by ***b*** from a quantifying profile pattern. If MT peak was selected as the reference standard, we can calculate the relative weight correction factor SPR/MT as $f_{SPR/MT}=\frac{A_{SPR}m_{MT}}{A_{MT}m_{SPR}}=1.024$ (RSD = 0.77%, n = 5) and OMT/MT as $f_{OMT/MT}=\frac{A_{OMT}m_{MT}}{A_{MT}m_{OMT}}=1.118$ (RSD = 0.48%, n = 5) according to the linear regression equations (seen in Table 2) of tri-marker. By taking those two factors into Eqs 1 and 2 respectively, we can get the changes for ***r*** and ***b*** within 1.2% that certainly can be ignored.

### Correlating LQTS with three marker Analyses

In this study, the alkaloid content in the ASF was accurately quantitated using the marker compounds (MT, OMT and SPR). However, the quantitative analysis requires the reference standards, calibration and more time. Even if quantitation is feasible when the reference standards are available, the quantitative results are only meaningful when acceptance criteria for the specific compounds exist. Unfortunately this is not the case for most of the herbal medicines. Therefore, fingerprint analysis becomes more critical if it is consistent with the quantitative results.

**Fig 5:**
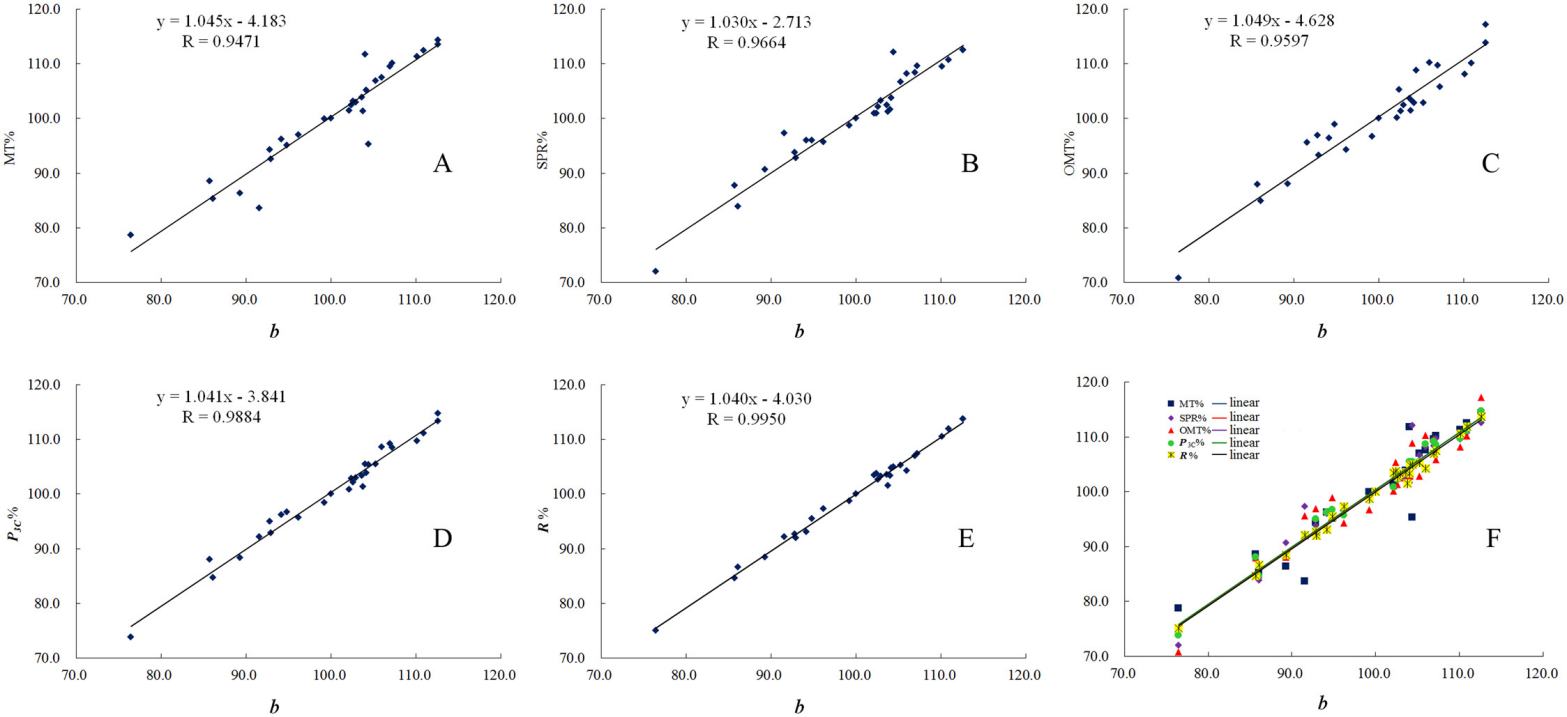
The linear regression plots of the content percentage (*P*_i_%) of the marker compounds vs. the linear quantitative similarity (*b*). (A) for MT, (B) for SPR, (C) for OMT, (D) for ***P***_3C_%, (E) for ***R***%, (F) for all the marker compounds, ***P***_3C_% and ***R***%.

As discussed in “Evaluating ASF quality by LQPM based on the fingerprint profiling” section, the linear quantitative similarity (***b***) is found to be more discriminating for the Alkaloids of Sophora flavescens. The relationship between LQTS (***b***) and the quantitative results of the marker compounds is further explored. First, the weight content of each marker compound in all the samples was averaged to generate its average content ($\bar{z}$), then the percentage of the *i*th marker content (***P***_*i*_%) for 27 samples (seen in S1 Table) was calculated out (shown in Eq 5) using a mass coefficient $f_{j}=\frac{m_{R}}{m_{j}}$. Secondly, the mean of ***P***_*i*_% values of the three marker compounds in each sample (***P***_*3C*_%) was calculated according to Eq 6. Meanwhile, ***R***% was calculated by Eq 4. Finally, ***P***_*i*_%, ***P***_3C_% and ***R***% of 27 samples were respectively plotted vs. ***b*** as shown in Fig 5. Linear regression shows that the correlation coefficients between ***P***_*i*_% and ***b*** are 0.9471, 0.9664 and 0.9597 for MT, SPR and OMT, respectively. In comparison, the correlation coefficient between ***P***_*3C*_% and ***b*** is significantly higher (0.9884). This indicates that ***b*** is highly correlated to the content of the marker compounds. It is not surprising that ***b*** is also highly correlated to the apparent content similarity (***R***%) as shown in Fig 5E, and the correlation coefficient between ***R***% and ***b*** reached the most excellent value of 0.9950. This demonstrates that LQTS (***b***) is a reliable substitute for the quantitative content of the marker compounds and is very effective in quantitatively evaluating the quality of herbal medicines. Therefore, the multiple markers analyses for quality control can be substituted by LQPM, which is simple, briefly, accurate and economic. ASF reference fingerprint contained 228.77mg/g of MT, 240.79 mg/g of SPR and 89.50 mg/g of OMT, and the total content of the tri-marker was 559.06 mg/g which was up to 70% (g/g) when water was taken out (please see the water content in below section ‘Overall distribution of basic substances in ASF samples’). So we can immediately calculate each content of the three marker compounds according to ***b***. From Table 3, the biggest error was not more than 3.5% for Δ*E*_1_ = ***R***% − ***P***_3*C*_%, Δ*E*_2_ = ***b*** − ***P***_3*C*_% and Δ*E*_3_ = ***b*** − ***R***%. In a word, LQTS (***b***) is a better way to determine the overall contents of ASF.

$$P_{i}\text{\%}=\frac{z_{i}}{\bar{z}}f_{j}\times 100\%$$ (5)

$$P_{3C}\%=\frac{1}{3}\sum_{i=1}^{3}P_{i}\times 100\%$$ (6)

### Correlating LQTS with LQLS

**Fig 6:**
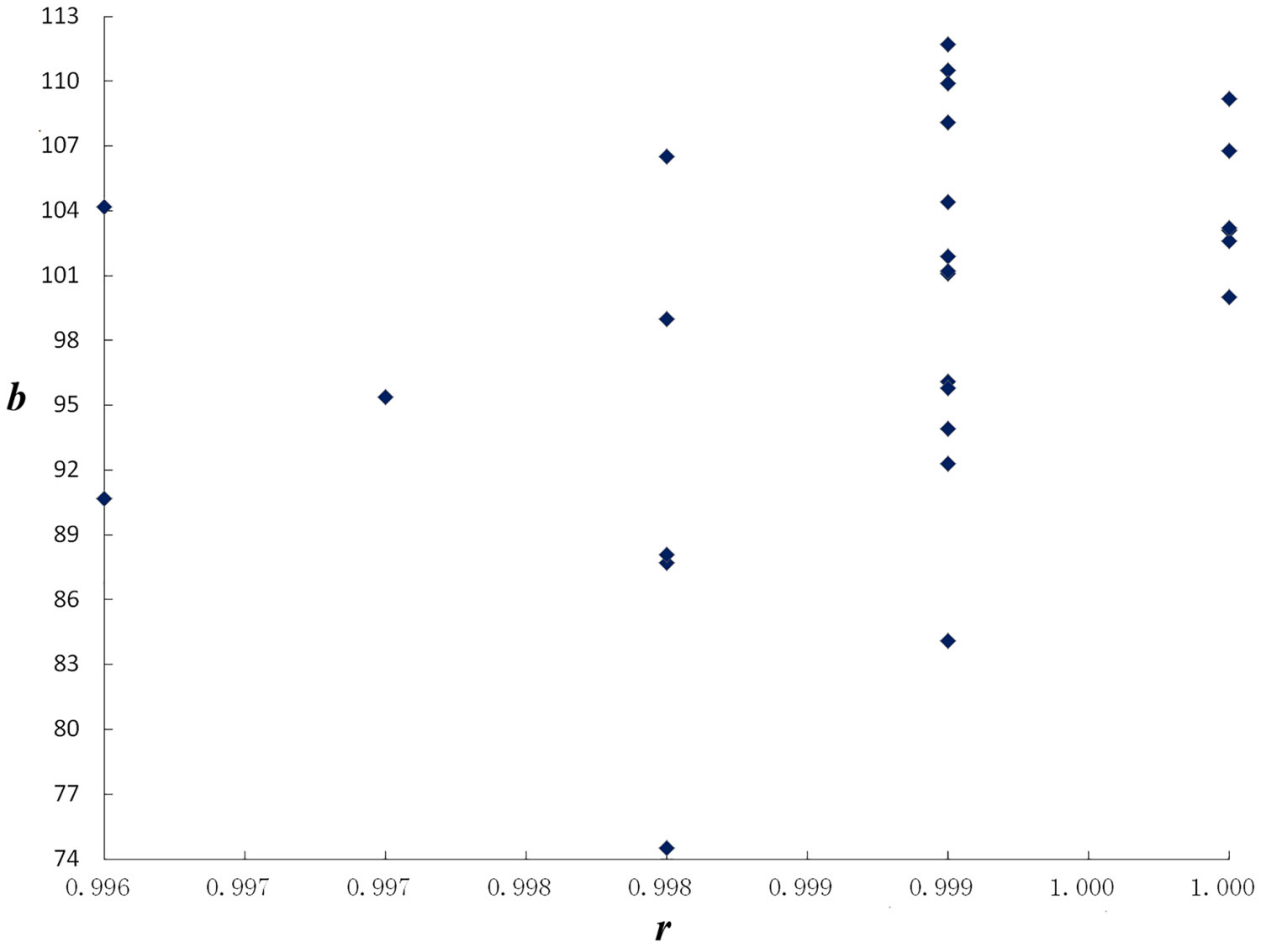
The plot of the quantitative similarity (*b*) vs. the qualitative similarity (*r*).

The reason why we use two similarities (LQLS and LQTS) to differentiate ASF quality based on chromatographic profiles is that there is no correlation between LQTS and LQLS of 29 profiles in Table 3. The Pearson correlation coefficient *R*^2^ = 0.1194 (n = 29) reflects that we cannot thoroughly evaluate quality of TCM in the accurate way by only using LQLS (***r*** all more than 0.996). So it is much necessary to use the second measure of LQTS (***b***) and it is the highest level evaluation for assessing fingerprint profiles of TCM. Fig 6 nearly displays an orthogonality correlation between ***r*** and ***b***, which indicates that the LQTS assess is more important than LQLS. Therefore LQPM is a novel fingerprint assessing method like SQFM [37], in which both LQLS and LQTS are used together for evaluating TCM quality, and a statistical error is used to monitor the method itself.

### The supplement quantifying method by the relative characteristic fingerprint (RCFP)

We can quantify all fingerprints by RCFP displayed in Fig 4B to give the same results as described in ‘Evaluating ASF quality by LQPM based on the fingerprint profiling’ section. Each fingerprint peak area was divided by the corresponding reference peak area of MT, so the relative peak area in RCFP was obtained. The parameter $A_{j}^{MT}$ is the area of MT in the *j*th sample and the parameter $A_{RFP}^{MT}$ is the area of MT in the reference fingerprint. When RCFP was assessed by LQPM, Eq 2 only need to be multiplied by the area correction factor of the reference peak (RP) $f_{RP}=\frac{A_{j}^{MT}}{A_{RFP}^{MT}}$, then the actual results can be revised back again. That is the reason why we could obtain the same results. The RCFP can also cancel the systematic differences in the different HPLC machines.

### Overall distribution of basic substances in ASF samples

Quality consistency of ASF samples were also monitored by analyzing the overall distribution of basic substances as shown in S2 Table. The average percent content of total alkaloid was 71.3%, as determined by an acid-base back-titration method in which 10.0 mL of sulfuric acid (0.05 mol/L) was added to the sample, and sodium hydroxide standard solution (0.1 mol/L) was used as a titrant to titrate the excessive sulfuric acid. The titration was carried out using methyl red as the indicator, with the titer expressed in terms of OMT (each 1 mL of sodium hydroxide is equivalent to 26.44 mg of OMT). The water content of all samples was determined to be 20.1% by using Karl Fischer titration with a titer of 3 mg H_2_O/mL. According to the above results, we can calculate the amount of total alkaloid content to be no less than 89.2% (calculated on the dried basis), which meets the requirement of no less than 70% in the national standards[30]. About 0.97% of total saponin was obtained by a colorimetric method at 520 nm by using vanillin-perchloric acid as the coloration system and oleanolic acid as the reference substance. About 0.69% of total amino acids was analyzed by visible spectrophotometry at 570 nm with ninhydrin as the developer and l-tyrosine as the reference substance. Method validations, including precision, stability, reproducibility and accuracy of recovery tests, were performed. The methods met the quantitative requirements as the recoveries were found to be between 98.5% and 100.7% (RSD ≤ 1.5%) and all corresponding RSD values did not exceed 0.5%. The 27 batches of ASF samples and the reference sample showed good consistency in the distribution of basic substances. But just like the quantitation of three marker compounds, none of these contents for certain total substances can discriminate the differences of the ASF samples.

## Conclusions

The present study demonstrated the use of the Linear Quantitative Profiling Method (LQPM) to evaluate the quality of the ASF samples based on the chromatographic fingerprints. All the test samples showed similar linear qualitative similarity (***r***) due to the predominant presence of three alkaloid compounds (MT, SPR and OMT) in the Alkaloids of Sophora flavescens; however, the linear quantitative similarity (***b***) were able to identify the difference among the different samples due to the variations in the quantitative content of the chemical components. Three marker alkaloids (MT, SPR and OMT) were also quantitated using the validated HPLC method to prove the effectiveness of quantitative profiling. Three samples were detected as qualitatively similar based on the quantitative data of the three marker compounds. The LQPM was able to correctly discover that one of the three samples (S18) actually had acceptable quality (Grade 1) considering its linear qualitative and quantitative similarity. In addition, two more samples were identified as dissimilar (S17 belonged to Grade 2 and S19 belonged to Grade 4) based on different linear quantitative similarities. This demonstrates that LQPM can be used to reliably evaluate the quality of ASF samples. The linear quantitative similarity was also shown to be highly correlated to the content of the marker compounds, indicating that quantitative analysis of the marker compounds may be substituted with LQPM based on the chromatographic fingerprints for the purpose of quantifying the multiple components of a complex sample system. Furthermore, once reference fingerprint (RFP) developed and the composition similarities calculated out, LQPM could employ the classical mathematical model to effectively quantify the multiple components of ASF samples without any chemical standard.

## Supporting Information

S1 Table The percentage of the marker contents (*P*_*i*_%) for ASF samples. (DOCX)

S2 Table The overall distribution of basic substances for ASF samples. (DOCX)
