# Correction: Linear Quantitative Profiling Method Fast Monitors Alkaloids of Sophora Flavescens that was Verified by Tri-marker Analyses

**DOI:** 10.1371/journal.pone.0168308

**Published:** 2016-12-12

**Authors:** Zhifei Hou, Guoxiang Sun, Yong Guo

In “The Linear Quantitative Profiling Method” section, there is an error in the first equation. Please see the complete, correct equation here:

$$r=\frac{\sum_{i=1}^{n}\left(x_{i}-\bar{x})(y_{i}-\bar{y}\right)}{\sqrt{\sum_{i=1}^{n}{\left(x_{i}-\bar{x}\right)}^{2}}\sqrt{\sum_{i=1}^{n}{\left(y_{i}-\bar{y}\right)}^{2}}}$$

In “The Linear Quantitative Profiling Method” section, there is an error in the second equation. Please see the complete, correct equation here:

$$b=\frac{n\sum_{i=1}^{n}x_{i}y_{i}-\sum_{i=1}^{n}x_{i}\sum_{i=1}^{n}y_{i}}{n\sum_{i=1}^{n}y_{i}^{2}-{\left(\sum_{i=1}^{n}y_{i}\right)}^{2}}\times \frac{m_{R}}{m_{j}}\times 100\%\approx \frac{\bar{x}}{\bar{y}}\times \frac{m_{R}}{m_{j}}\times 100\%=R\times \frac{m_{R}}{m_{j}}\times 100\%$$
