# Supplementary material for: Linear Quantitative Profiling Method Fast Monitors Alkaloids of Sophora Flavescens That Was Verified by Tri-Marker Analyses
Source: PLoS One. 2016 Aug 16;11(8):e0161146. doi: 10.1371/journal.pone.0161146 (PMC4987015; doi:10.1371/journal.pone.0161146)
Supplement: S1 Table — (DOCX) [file pone.0161146.s001.docx]

**S1 Table. The percentage of the marker contents (*P_i_*%) for ASF samples**

| **No.** | **S1** | **S2** | **S3** | **S4** | **S5** | **S6** | **S7** | **S8** | **S9** | **S10** | **S11** | **S12** | **S13** | **S14** | **S15** |
| --- | --- | --- | --- | --- | --- | --- | --- | --- | --- | --- | --- | --- | --- | --- | --- |
| MT% | 95.1 | 85.3 | 102.9 | 103.2 | 101.5 | 111.3 | 102.4 | 105.2 | 112.5 | 106.9 | 103.9 | 113.5 | 110.1 | 114.3 | 97.0 |
| SPR% | 96.0 | 83.9 | 103.3 | 102.1 | 100.9 | 109.5 | 100.9 | 103.7 | 110.7 | 106.7 | 102.4 | 112.6 | 109.6 | 112.6 | 95.7 |
| OMT% | 98.9 | 84.9 | 102.5 | 101.3 | 100.1 | 108.2 | 105.3 | 102.9 | 110.1 | 102.8 | 103.7 | 113.8 | 105.8 | 117.2 | 94.3 |
| **No.** | **S16** | **S17** | **S18** | **S19** | **S20** | **S21** | **S22** | **S23** | **S24** | **S25** | **S26** | **S27** | **RFP** | **RS** |  |
| MT% | 109.6 | 83.6 | 95.3 | 78.7 | 111.8 | 107.5 | 101.3 | 96.2 | 92.6 | 94.3 | 88.6 | 86.3 | 100.0 | 99.9 |  |
| SPR% | 108.4 | 97.3 | 112.1 | 72.0 | 101.7 | 108.2 | 101.2 | 96.0 | 92.8 | 93.8 | 87.7 | 90.7 | 100.0 | 98.7 |  |
| OMT% | 109.7 | 95.6 | 108.8 | 70.8 | 103 | 110.2 | 101.5 | 96.4 | 93.3 | 96.9 | 88.0 | 88.1 | 100.0 | 96.8 |  |
