# Supplementary material for: Linear Quantitative Profiling Method Fast Monitors Alkaloids of Sophora Flavescens That Was Verified by Tri-Marker Analyses
Source: PLoS One. 2016 Aug 16;11(8):e0161146. doi: 10.1371/journal.pone.0161146 (PMC4987015; doi:10.1371/journal.pone.0161146)
Supplement: S2 Table — (DOCX) [file pone.0161146.s002.docx]

**S2 Table. The** [**overall distribution**](javascript:showjdsw('showjd_0','j_0')) **of basic substances for ASF samples**

| **No.** | **Total alkaloid** | **water** | **Total saponin** | **Total amino acids** |
| --- | --- | --- | --- | --- |
| **Average percent content for 27 ASF samples** | 71.33% | 20.07% | 0.97% | 0.69% |
| **RS** | 70.27% | 20.20% | 0.96% | 0.70% |
